# Mycolic Acid Modification by the *mmaA4* Gene of *M. tuberculosis* Modulates IL-12 Production

**DOI:** 10.1371/journal.ppat.1000081

**Published:** 2008-06-06

**Authors:** Dee N. Dao, Kari Sweeney, Tsungda Hsu, Sudagar S. Gurcha, Ivan P. Nascimento, Dan Roshevsky, Gurdyal S. Besra, John Chan, Steven A. Porcelli, William R. Jacobs

**Affiliations:** 1 Howard Hughes Medical Institute, Albert Einstein College of Medicine, Bronx, New York, United States of America; 2 Department of Microbiology and Immunology, Albert Einstein College of Medicine, Bronx, New York, United States of America; 3 School of Biosciences, The University of Birmingham, Edgbaston, United Kingdom; 4 Instituto Butantan, Biotecnologia Molecular IV, São Paulo, São Paulo, Brazil; 5 Department of Medicine, Albert Einstein College of Medicine, Bronx, New York, United States of America; University of Washington, United States of America

## Abstract

*Mycobacterium tuberculosis* has evolved many strategies to evade elimination by the host immune system, including the selective repression of macrophage IL-12p40 production. To identify the *M. tuberculosis* genes responsible for this aspect of immune evasion, we used a macrophage cell line expressing a reporter for IL-12p40 transcription to screen a transposon library of *M. tuberculosis* for mutants that lacked this function. This approach led to the identification of the *mmaA4* gene, which encodes a methyl transferase required for introducing the distal oxygen-containing modifications of mycolic acids, as a key locus involved in the repression of IL-12p40. Mutants in which *mmaA4* (*hma*) was inactivated stimulated macrophages to produce significantly more IL-12p40 and TNF-α than wild-type *M. tuberculosis* and were attenuated for virulence. This attenuation was not seen in IL-12p40-deficient mice, consistent with a direct linkage between enhanced stimulation of IL-12p40 by the mutant and its reduced virulence. Treatment of macrophages with trehalose dimycolate (TDM) purified from the Δ*mmaA*4 mutant stimulated increased IL-12p40, similar to the increase observed from Δ*mmaA*4 mutant-infected macrophages. In contrast, purified TDM isolated from wild-type *M. tuberculosis* inhibited production of IL-12p40 by macrophages. These findings strongly suggest that *M. tuberculosis* has evolved *mmaA4*-derived mycolic acids, including those incorporated into TDM to manipulate IL-12-mediated immunity and virulence.

## Author Summary

Currently, one-third of the world's population has tuberculosis (TB). TB, an ancient foe, has reemerged to become a threat to global public health. A central problem in TB research is to investigate why the host immune system cannot sterilize the infection caused by the bacterium *Mycobacterium tuberculosis*. Interleukin-12 (IL-12), a molecule produced by macrophages in response to pathogens, plays an important role in orchestrating sterilizing immunity. However, *M. tuberculosis* has evolved mechanisms that block IL-12 production and thereby assist the bacterium in establishing chronic infection. We discovered that mutation of the mycobacterial *mmaA4* gene, which controls the chemical modification of complex lipids of *M. tuberculosis* called mycolic acids, renders the bacterium unable to block IL-12 production. Mycolic acids incorporated into a secreted bacterial molecule called trehalose dimycolate (TDM) from *M. tuberculosis* had the ability on their own to suppress the production of IL-12 by activated macrophages; we also showed that TDM from the *mmaA4* mutant of *M. tuberculosis* is attenuated for suppression. Our results identify a critical part of the genetic basis and mechanism for an important immune evasion function in *M. tuberculosis*, and should contribute to the design of future vaccines and immunotherapies for disease caused by this pathogen.

## Introduction

Tuberculosis (TB) is the second leading cause of death from an infectious disease worldwide [1],[2]. *Mycobacterium tuberculosis* is well adapted to the human host, and possesses a variety of mechanisms that promote immune evasion and thereby permit latent infection in the presence of host innate and adaptive immune responses [3],[4]. This latent reservoir of *M. tuberculosis* can eventually develop into active disease when the host immune system is compromised by any of a variety of factors, the most common of which are aging, malnutrition, and concurrent infection by HIV [5]–[7]. Currently, the attenuated *M. bovis* strain, BCG, is the only vaccine available for routine human immunization. It has had little if any impact on the increasing global prevalence of TB, in spite of having been administered to more than a billion people [8]. Thus, work on developing new and more immunogenic vaccine candidates is crucial and requires advances in our understanding of the host-pathogen interaction.

Because phagocytic cells recognize microbes before the development of specific immunity, the macrophage response to the infection is critical for the initial local containment of infection and the subsequent development of adaptive immunity. The cytokine profile produced by macrophages and other antigen-presenting cells within the first days or weeks following infection can define the type of host immunity induced, and thereby determine its effectiveness for controlling the microbial infection [9]. A critical cytokine in the control of intracellular infections is interleukin-12 (IL-12), which is produced mainly by macrophages and dendritic cells [10]. Members of the IL-12 family, including IL-12p80, IL-12p70, and IL-23, are central players in various arms of early nonspecific innate immune resistance and subsequent antigen-specific adaptive immune responses to *M. tuberculosis* [11]–[13]. These cytokines are dimers that all share the common IL-12p40 subunit in association with a different partner, and each has a different immunoregulatory role during various stages of the immune response to intracellular pathogens [14]. For example, during innate immune response, macrophages release IL-12p80, which is a homodimer of p40 subunits, upon initial infection to stimulate local recruitment of more macrophages. The IL-12p70 cytokine, a heterodimer of p35 and p40 subunits, induces macrophage bactericidal activity and also proliferation, cytolytic activity, and IFNγ production by NK cells during the early innate phase of the immune response. During the induction of the adaptive response, IL-12p70 produced by macrophages and dendritic cells plays a central role in polarizing T helper type 1 (Th1) differentiation [10]. In addition to IL-12p70, IL-12p80 also has a role in initiating adaptive immunity [15]. Finally, maintenance and recall responses of immunological memory require both IL-12p70 and IL-23 (a dimer of p40 and p19) [16].

It is known that mice and humans with mutations in the IL-12p40 or IL-12 receptor genes are highly susceptible to mycobacterial infection, highlighting the importance of this family of cytokines in resistance to infection with these bacteria [17], [18]. However, even in a host that is genetically normal with respect to its IL-12 axis, virulent *M. tuberculosis* can evade eradication. This may be explained by the fact that resistance versus susceptibility to intracellular pathogens is often determined by a delicate balance between the cytokines that initiate and those that inhibit immunity. Studies of the mechanisms of immune evasion by *M. tuberculosis* have revealed that one of the strategies used by the tubercle bacillus to resist eradication is to actively repress macrophage production of IL-12p40 while stimulating secretion of IL-10, an inhibitor of IL-12 mediated-immunity [19],[20]. This immune evasion mechanism parallels that proposed for *Leishmania* and *Toxoplasma*, another highly persistent intracellular pathogen [21],[22].

Given previous findings on the ability of IL-12 to enhance protective immunity and extend survival in mice and humans infected with *M. tuberculosis* [12],[23],[24], and the discovery that *M. tuberculosis* represses IL-12p40 production, we hypothesized that *M. tuberculosis* actively dampens the production of IL-12p40 cytokine in infected macrophages. To identify the mechanisms and effector molecules responsible for this, we screened a transposon library of *M. tuberculosis* mutants using a macrophage cell line expressing a reporter gene for monitoring IL-12p40 expression. This identified a major role in IL-12p40 repression for the *mmaA4* (methoxy mycolic acid synthase 4) gene, which encodes the methyl transferase that introduces oxygen-containing modifications of cell wall mycolic acids. Mutants in which *mmaA4* was inactivated induced more IL-12p40 from murine macrophages, and were attenuated for virulence in mice. The attenuation of virulence was reversed in IL-12p40-deficient mice, indicating that this attenuation depended on IL-12p40-mediated immunity. Furthermore, the abundant surface and secreted glycolipid trehalose 6,6′-dimycolate (TDM) was identified as an effector molecule for the repression of IL-12p40 production by *M. tuberculosis*. However, purified TDM from the Δ*mmaA4* mutant, which contained mycolates that were devoid of distal oxygen-containing modifications, stimulated markedly increased production of IL-12p40 and TNF-α compared to the levels resulting from stimulation with TDM from wild type *M. tuberculosis*. Our data identify the role of *mmaA4*-dependent mycolic acid modifications in the repression of IL-12p40 production, thus establishing part of the genetic and mechanistic basis for an important aspect of the immune evasion strategy of *M. tuberculosis*.

## Results

### Isolation of *M. tuberculosis* mutants defective in repression of IL-12p40 production

**Figure 1:**
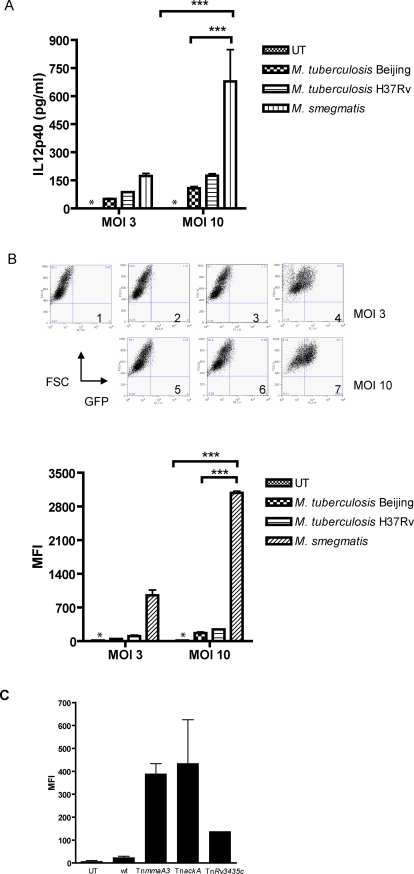
Macrophages infected with virulent strains of *M. tuberculosis* produced significantly less IL-12p40 than avirulent strain and candidate mutants. (A) BALB/c bone marrow-derived macrophages were infected at an MOI of 3 or an MOI of 10 with virulent clinical (Beijing/W (HN878)) or laboratory (H37Rv) strains of *M. tuberculosis,* with the avirulent mycobacterial species *M. smegmatis*, or left untreated (UT). IL-12p40 from conditioned media harvested for 16 to 24 hr was analyzed by ELISA. At an MOI of 10, *M. smegmatis* induced significantly more IL-12p40 than virulent *M. tuberculosis* Beijing and *M. tuberculosis* H37Rv (***, *p*<0.001; two-way ANOVA, Bonferroni post-tests). Values are the means±SD for triplicate samples and are representative of 3 separate experiments. (*), below limit of detection. (B) Evaluation of IL-12p40 promoter activity in macrophages infected with virulent or avirulent strains of mycobacteria. The -800+55 IL-12p40-GFP Raw 264.7 cell line was infected at an MOI of 3 or 10 with virulent *M. tuberculosis* Beijing/W (HN878), H37Rv, or *M. smegmatis,* or left untreated (UT). Cells were harvested for 16 to 24 hr following infection to measure GFP expression by flow cytometry. Top: sample dot plots are shown for infections at MOI of 3 or 10, as indicated. (1) Uninfected; (2) and (5), *M. tuberculosis* Beijing/W (HN878); (3) and (6), *M. tuberculosis* H37Rv; (4) and (7), *M. smegmatis*. Bottom: graph of mean fluorescence intensity (MFI) values for GFP expression. ***, *p<*0.001 (two-way ANOVA, Bonferroni post-tests). Values are the means±SD of triplicate samples and are representative of 3 separate experiments. *, below limit of detection. (C) Transposon insertion mutants of *M. tuberculosis* that induced increased expression of IL-12p40 in macrophage reporter cell line. Secondary screen of expanded cultures of candidate transposon mutants. Transposon (Tn) insertion mutants in the indicated genes were used to infect the −800+55 IL-12p40-GFP Raw 264.7 cell line at an MOI of 10. Flow cytometry analysis was used to analyze GFP expression of infected macrophages. One-way ANOVA analysis showed that the difference in mean fluorescence intensity (MFI) values for the entire set was statistically significant (*p*<0.05). Values are the means±SD of triplicate samples and are representative of 2 separate experiments.

To screen for mutants of *M. tuberculosis* that were defective in repression of IL-12p40 production, we generated a macrophage reporter cell line to monitor IL-12p40 expression. Previously, we described a Raw 264.7 murine macrophage cell line containing a stably integrated construct of the minimal IL-12p40 promoter fused to GFP [25]. Since the *cis* elements required for regulation of the IL-12p40 promoter in response to *M. tuberculosis* infection are not known, we engineered another Raw 264.7 line containing a stable integration of the full-length IL-12p40 promoter fused to GFP. Flow cytometry analysis showed that GFP was not transcribed at baseline in this macrophage line, but was induced upon treatment with lipopolysaccharide (LPS) or infection with *E. coli* (data not shown). Following with mycobacterial strains and species that varied in their virulence, we observed by flow cytometry the levels of GFP induction that followed a pattern similar to what was observed using a capture ELISA to quantitate IL-12 p40 levels in supernatants of similarly infected bone marrow-derived macrophages (Figs. 1A and 1B). Both the GFP expression of the reporter macrophage cell line and supernatant levels of IL-12p40 in primary macrophage cultures confirmed that the avirulent *M. smegmatis* strain was a robust inducer of IL-12p40 production. In contrast, the virulent clinical (Beijing HN878) and laboratory (H37Rv) strains of *M. tuberculosis* induced only minimally detectable transcription and secretion of this cytokine, consistent with previously reported results [26],[27].

To explore the hypothesis that the low levels of IL-12p40 produced by macrophages infected with *M. tuberculosis* resulted from active inhibition of IL-12p40 transcription, we used the reporter cell line to screen for mutant bacilli that had lost this function. Since a 2-14-fold difference in GFP expression could be detected by this reporter cell line (Fig. 1B and data not shown), the system was sensitive enough to allow for detection of incremental changes in the promoter activity. A library of transposon insertion mutants of the sequenced *M. tuberculosis* H37Rv strain was created using the Himar-1 transposon and arrayed as individual clones in 96-well plates. We used this transposon to generate *M. tuberculosis* mutants because it inserts randomly into frequently-occurring TA dinucleotides [28]. Approximately 2880 transposon mutants were infected individually into the macrophage reporter cells, and screened by using a fluorimetric assay to identify GFP expression that was greater than a baseline established by wild type H37Rv infection. A primary screen identified three mutants of interest, which were found by sequencing of the transposon insertion sites to have interruptions in open reading frames *Rv0643c*, *Rv0409,* and *Rv3435c*. Among these candidate genes, *Rv0643c* is the most extensively characterized, and is annotated as the *methoxy mycolic acid synthase 3* (*mmaA3*) gene. *Rv0409* is also known as *ackA* and encodes a putative acetate kinase, while *Rv3435c* encodes a predicted transmembrane protein of unknown function.

### Analysis of transposon mutants and identification of *mmaA4* as a locus involved in repression of IL-12p40 responses in macrophages

A secondary screen using analysis of the infected macrophage reporter cells by FACS confirmed that the three mutants identified in the primary screening reproducibly stimulated enhanced GFP expression, with the clone bearing a transposon insertion in the *mmaA3* gene showing the highest GFP expression (Fig. 1C). In a tertiary screen for IL-12p40 production by ELISA of supernatant levels from bone marrow-derived macrophages infected with these mutants, the *mmaA3* mutant again showed increased IL-12p40 production. In contrast, the *ackA* and *Rv3435c* mutants showed inconsistent results in the tertiary screening with primary macrophages (data not shown), and thus were not analyzed further. To confirm and further evaluate the role of *mmaA3* in modulating IL-12p40 expression, the *mmaA3* gene was deleted from *M. tuberculosis* H37Rv by specialized transduction [29]. Since the transcriptional regulation of the *mmaA4* gene immediately downstream of *mmaA3* could also have been compromised by the transposon insertion in *mmaA3*, we also generated and studied an *M. tuberculosis* strain with deletion in the *mmaA4* gene.

Using an ELISA to quantitate IL-12p40 in the medium of bone marrow-derived macrophage cultures infected with either the Δ*mmaA3* or Δ*mmaA4* mutants, we observed that infection of macrophages with the Δ*mmaA3* mutant showed variable increases in IL-12p40 production (data not shown). In addition, when grown on agar plates, the Δ*mmaA3* mutant showed a mixture of both rough and smooth colony morphologies (data not shown), indicating that phenotypic switching between these two morphologies was occurring frequently during culture and may have accounted for the variable effects on IL-12p40 induction. Because of this potentially confounding variable and the inconsistent effects on IL-12p40 production, no additional studies were pursued with the Δ*mmaA3* mutant and all subsequent work focused on the Δ*mmaA4* mutant.

**Figure 2:**
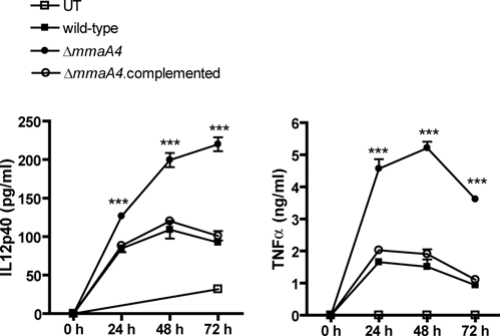
Increased induction of IL-12p40 and TNF-α by the Δ*mmaA4 M. tuberculosis* mutant in bone marrow-derived macrophages. Bone marrow-derived macrophages from BALB/c mice were infected with wild type *M. tuberculosis* H37Rv, the Δ*mmaA4* mutant, or the complemented Δ*mmaA4* strain at an MOI of 10, or left untreated (UT). Conditioned media from macrophage cultures were harvested at 24, 48, and 72 hr post-infection. IL-12p40 and TNF-α production were determined by ELISA. ***, *p*<0.001 (two-way ANOVA, Bonferroni post-tests). Values are the means±SD of triplicate samples and are representative of 3 separate experiments.

In contrast to Δ*mmaA3* infected macrophages, bone marrow-derived macrophages infected with the Δ*mmaA4* mutant showed a reproducible increase in both IL-12p40 and TNF-α production, compared to wild type *M. tuberculosis*. This phenotype was reversed by complementation using chromosomal insertion of a single copy of the wild type *mmaA4* gene (Fig. 2). IL-12p40 and TNF-α production in macrophages infected with the Δ*mmaA4* mutant increased over time, and similar cytokine increases were observed for bone marrow-derived macrophages from two different mouse strains (i.e., BALB/c in Fig. 2, and C57BL/6 in Fig. S1). It is known that dendritic cell also produce IL-12p40 following infection with *M. tuberculosis* [19],[30]. To examine dendritic cell responses, bone marrow-derived dendritic cells were infected with the parental, mutant, or complemented strain. Dendritic cells infected with the Δ*mmaA4* mutant produced copious amounts of IL-12p40. This robust production of IL-12p40 was also observed for dendritic cells infected with wild type or complemented strain (Fig. S2A). Thus, the Δ*mmaA4* mutant selectively effects macrophage cytokine production.

**Figure 3:**
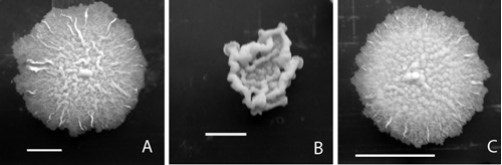
Changes in colonial morphology following deletion of *mmaA4* gene. Colonial morphologies of bacteria grown on 7H10 plates containing 0.05% Tween-80. Photographs were taken after 2 months of growth. (A) wild type *M. tuberculosis* H37Rv (B) Δ*mmaA4* mutant (C) Δ*mmaA4* complemented. Bar is 1 cm.

The Δ*mmaA4* mutant maintained stable colony morphology with routine passage, showing a smooth colony morphology with ruffled edges when plated on media containing the detergent Tween-80 (Fig. 3). This was distinctly different from the rough colony morphology observed with similarly cultured wild type *M. tuberculosis*. This difference in colony morphology was reversed by complementation of the Δ*mmaA4* mutant (Fig. 3). The growth rate of the Δ*mmaA4* mutant in liquid culture was equivalent to that of wild type (data not shown).

Previous studies of mycolic acids synthesized by the *mmaA4* mutant (also known as *hma*, for *hydroxyl mycolic acid synthase*) or by using the expression of the *M. tuberculosis mmaA4* gene in *M. smegmatis*, provided strong evidence that the enzyme encoded by this locus is responsible for introducing the methyl and adjacent hydroxy groups on the distal meromycolate chain of the common precursor for methoxy- and keto-mycolic acids [31],[32]. This was also supported by analysis of mycolic acid and major extractable lipids in the wild type, Δ*mmaA4* mutant, and complemented strains in this study (Figs. S3 and S4). Additionally, our finding that growth of the Δ*mmaA4* mutant in the presence of Tween-80 caused changes in colony morphology was consistent with a significant alteration in the lipid composition of the cell wall [33], which would be expected given that mycolates are among the most abundant cell wall lipids.

### Increased induction of IL-12p40 and TNF-α by trehalose 6,6′ -dimycolate (TDM) from the Δ*mmaA4* mutant

**Figure 4:**
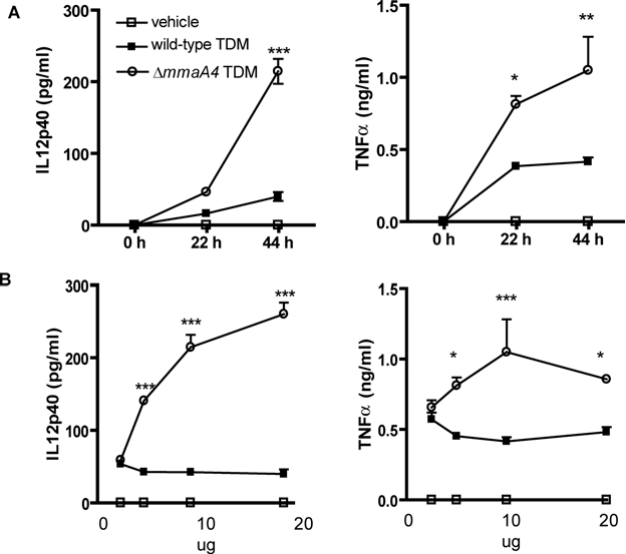
Purified TDM from Δ*mmaA4* mutant stimulated macrophages to produce IL-12p40. (A) Bone marrow-derived macrophages were treated with 5 µg of purified *M. tuberculosis* TDM (wild type TDM) or mutant TDM (Δ*mmaA4* TDM). Cytokines in conditioned media were harvested at 22 and 44 hr post-TDM treatment. Supernatants were analyzed for the presence of IL-12p40 and TNF-α by ELISA. Vehicle treatment is the solvent in which the TDM was dissolved. Statistically significant differences for Δ*mmaA4* TDM compared to wild type TDM are indicated by asterisks as follows: *, *p*<0.05 (_*_); **, *p*<0.01; ***, *p*<0.001 (two-way ANOVA, Bonferroni post-tests). Values are the means±SD of triplicate samples and are representative of 3 separate experiments performed on 2 independent batches of purified TDMs. (B) Dose responses to TDM purified from wild type *M. tuberculosis* H37Rv (wild type TDM) or Δ*mmaA4* mutant (*mmaA4* TDM). Bone marrow-derived macrophages were treated with varying doses of TDM purified from wild type or Δ*mmaA4* mutant *M. tuberculosi*s. Conditioned media were assayed for cytokines at 44 hr by ELISA. Symbols as in (A); values are the means±SD of triplicate samples and are representative of 2 separate experiments performed on 2 independent batches of purified TDMs.

A small but significant quantity of mycolic acids are found noncovalently associated with cell wall glycolipids; the most abundant of these is TDM [34]. Since TDMs are released into the cytoplasm of macrophages infected with mycobacteria, this suggested that the alterations in cytokine production seen with the Δ*mmaA4* mutant might be due to changes in the mycolic acids incorporated into its TDMs [35]. This possibility was studied by comparing the cytokine responses of macrophages to TDMs purified from wild type and from Δ*mmaA4* mutant *M. tuberculosis*. Time course and dose response studies with purified TDM from either wild-type or the Δ*mmaA4* mutant clearly showed that the Δ*mmaA4* TDM mutant induced significantly more IL-12p40 and TNF-α than did wild type TDM (Fig. 4A). IL-12p40 production was detected at 22 hr in conditioned media from macrophages that were treated with Δ*mmaA4* TDM, and further increased 4-fold by 44 hr. TNF-α was detected at 22 hr, with no further increase thereafter (Fig. 4B). In contrast, after 22 to 44 hr of incubation with wild type TDM, production of IL-12p40 remained constant and was substantially less then that stimulated by Δ*mmaA4* TDM (Fig. 4A). By comparison, IL-12p40 and TNF-α production increased with the addition of increasing amounts of Δ*mmaA4* TDM to the cultures, whereas wild type TDM did not show a dose dependency over the range of TDM concentrations tested (Fig. 4B). Similar differences in cytokine production by macrophages treated with trehalose monomycolate (TMM) from the mutant and wild type strains were observed (Fig. S5). These findings indicated that the increased macrophage cytokine production following infection with Δ*mmaA4* mutant bacteria could potentially have been mediated by the release of modified TDMs.

### Inhibition of IL-12p40 production in macrophages by TDM of wild type *M. tuberculosis*

**Figure 5:**
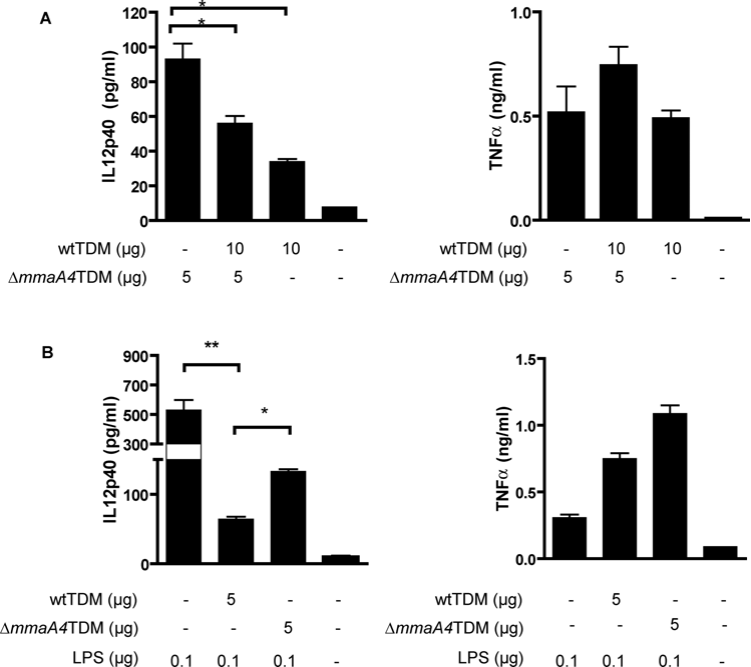
Purified wild type *M. tuberculosis* TDM inhibits macrophage IL-12p40 induction. (A) Wild type *M. tuberculosis* H37Rv TDM (wtTDM) dampens IL-12 production induced by ΔmmaA4 mutant TDM. Bone marrow-derived macrophages were incubated with Δ*mmaA4* TDM alone or with a mixture of purified wild type TDM and Δ*mmaA4* TDM. IL-12p40 and TNF-α were measured in culture supernatants by ELISA. *, *p*<0.05 (one-way ANOVA, Bonferroni post-tests). Values are the means±SD of triplicate samples and are representative of two separate experiments performed on at least two independent batches of purified TDM. (B) *M. tuberculosis* TDM (wtTDM) dampens IL-12 production induced by LPS. IL-12 p40 and TNF-α accumulation was measured by ELISA in supernatants of bone-marrow derived macrophages stimulated with LPS alone, or with LPS combined with either wild type TDM or Δ*mmaA4* TDM. *, *p*<0.05; **, p<0.01 (one-way ANOVA, Bonferroni post-tests). Values are the means±SD of triplicate samples and are representative of 3 separate experiments performed on at least 2 independent batches of purified TDM.

The differences in cytokine production observed for macrophages treated with TDM-containing oxygenated mycolic acids versus mutant TDMs lacking oxygenated mycolic acids suggested a possible inhibitory effect of the oxygenated mycolic acids on IL-12p40 responses. To test this, we analyzed the effects of mixing wild type TDM and Δ*mmaA4* TDM on macrophage cytokine responses. This experiment revealed that combining the two TDMs led to significant inhibition of the IL-12p40 response stimulated by the mutant TDM alone (Fig. 5A, left). Interestingly, this apparent inhibition of cytokine production was not observed for TNF-α production, which was actually slightly enhanced when the TDMs from wild type and mutant bacteria were combined (Fig. 5A, right).

To further assess this apparent repression of IL-12p40 production by wild type but not by Δ*mmaA4* mutant TDMs, we examined the impact of each on IL-12p40 production in response to *E. coli* lipopolysaccharide (LPS). This analysis showed that TDMs from both sources reduced the LPS-induced IL-12p40 response, but that wild type TDM was significantly more potent in this regard than was Δ*mmaA4* TDM (Fig. 5B, left). In contrast, we observed that neither type of TDM repressed the LPS-induced TNF-α production (Fig. 5B, right). In fact, a trend was observed of increased stimulation of TNF-α production by the mutant, as compared to the wild type, TDM. Taken together, these data suggested that the TDM of wild type *M. tuberculosis* was a significant mediator of IL-12p40 repression in macrophages, whereas the TDMs of the Δ*mmaA4* mutant, which lack keto- and methoxy-mycolates, were much less potent in this regard. In addition, it was apparent from these results that wild type TDMs possessed both weak stimulatory and strong inhibitory activity for macrophage production of IL-12p40, whereas the Δ*mmaA4* TDM was significantly more stimulatory and had reduced capacity to inhibit IL-12p40 responses.

### IL-12p40 dependent attenuation of virulence of the Δ*mmaA4* mutant in mice

**Figure 6:**
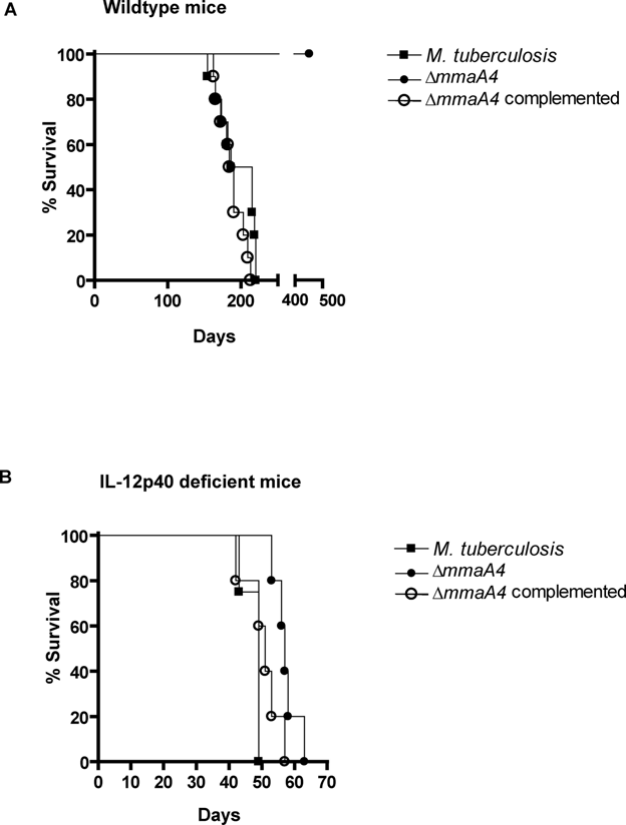
Requirement of IL-12p40 for attenuation of virulence of *M. tuberculosis* Δ*mmaA4*. (A) Survival of mice infected with wild-type *M. tuberculosis* H37Rv, the Δ*mmaA4* mutant, or the complemented Δ*mmaA4* mutant. Ten mice each were infected with approximately 100 CFU via the aerosol route and their survival times were recorded. The difference between the survival curves of Δ*mmaA4* infected mice and that of wild type or complemented mutant-infected groups is highly significant (Logrank test *p*<0.0001). Results are representative of 2 independent experiments. (B) Survival of IL-12p40-deficient mice infected with wild-type *M. tuberculosis* H37Rv, the Δ*mmaA4* mutant, or the Δ*mmaA4* complement. Five mice were infected for the mutant and the complemented groups, and four for the wild-type group, with approximately 125 CFU via the aerosol route, and their survival times were recorded. There was no significant difference in survival curves for Δ*mmaA4-*infected animals compared to wild type- or complemented mutant-infected animals (Logrank test *p = *0.08). Survival study shown is from one of two independent experiments.

The increased IL-12p40 and TNF-α observed in macrophages infected with Δ*mmaA4 M. tuberculosis* or treated with its TDM could be hypothesized as potentially having either of two opposing outcomes *in vivo*. The increases in these cytokines could either induce a protective immune response and improved host survival, or could lead to a deleterious exacerbation of immunity with tissue damage, contributing to poor outcome. To distinguish between these two possibilities, we infected immunocompetent C57BL/6 mice with the Δ*mmaA4* mutant and compared their survival rate to that of mice infected with wild type (H37Rv) *M. tuberculosis* or the complemented Δ*mmaA4* strain. As shown in Fig. 6A, all mice infected with wild type *M. tuberculosis* died by approximately 225 days post-infection. In contrast, all mice infected with the Δ*mmaA4* mutant survived 450 days post-challenge, while complementation of Δ*mmaA4* mutation restored virulence to the mutant.

To examine whether the attenuation in virulence observed for mice infected with the Δ*mmaA4* mutant was dependent on IL-12p40, we assessed the survival rate of IL-12p40 deficient mice infected with wild type *M. tuberculosis*, the Δ*mmaA4* mutant, or the complemented mutant. IL-12p40-deficient mice infected with wild type *M. tuberculosis* or the complemented mutant strain all died between 45 and 60 days. Noticeably, IL-12p40-deficient mice infected with the Δ*mmaA4* mutant survived only slightly longer (p = 0.08 by Logrank test, compared to wild type *M. tuberculosis* infected animals), with all of these animals dying by 62 days post-infection (Fig. 6B). This finding was consistent with the conclusion that the attenuation of the Δ*mmaA4* mutant *in vivo* was dependent on the presence of a normally functioning IL-12p40 gene as well as on the ability of this mutant to elicit a more robust IL-12 response. This *in vivo* study provided additional support for the view that the repression of IL-12p40 by mycolic acids with oxygen-containing modifications plays a major role in immune evasion that leads to the virulence of *M. tuberculosis*.

## Discussion

*M. tuberculosis* has evolved strategies to evade the antimicrobial effects of IL-12-induced immunity, including selective repression of IL-12p40 expression in macrophages [19],[20]. To identify the factor(s) involved in this evasion strategy, we screened for mutants that induced IL-12p40, and identified the *M. tuberculosis mmaA4* gene as a key locus involved in modulation of IL-12p40. We demonstrated that infection of macrophages with the Δ*mmaA4* mutant of *M. tuberculosis* H37Rv resulted in production of more IL-12p40. In addition, our results strongly suggest that this enhanced induction of IL-12p40 could be mediated by the mycolate-containing glycolipid TDM, which is known to be secreted as a potential immunomodulator into the cytosol of infected macrophages. Consistent with this view, we also showed that TDM from wild type *M. tuberculosis* repressed macrophage IL-12p40 production. To our knowledge, this is the first demonstration of such inhibitory activity for TDMs of *M. tuberculosis*, and also the first data to implicate the methoxy- and keto- modifications of the mycolates in TDM in the expression of this inhibitory activity on macrophages.

Consistent with findings reported in the literature that *M. tuberculosis* does not repress IL-12 production in dendritic cells, we found that dendritic cells produced similar amounts of IL-12, whether infected with the wild type, the complemented strain, or the Δ*mmaA4* mutant. Additionally, dendritic cells treated with TDM produced levels of IL-12 comparable to those resulting from LPS treatment. Thus, the *mmaA4* mutation has a selective effect on macrophages. It is interesting to note that suppression of IL-12 production in macrophage, but not dendritic, cells as an immune evasion mechanism has also been observed for other well-characterized persistent intracellular pathogens, such as, *Leishmania* and *Toxoplasma* [21],[22].

Two independent reports have shown that constitutive expression of the IL-12p40 gene in mice did not improve host immunity against *M. tuberculosis* [36],[37]. Nevertheless, IL-12 is necessary and sufficient for achieving normal levels of protective host immunity against *M. tuberculosis* and other mycobacteria [11],[12],[24]. This disparity between a protective response when IL-12 is produced at the time of infection and a lack of enhanced immunity when IL-12 is constitutively expressed, underscores the importance of temporal and spatial regulation of IL-12p40 expression during infection. Thus, any perturbation of such regulation, such as constitutive and generalized expression, or *M. tuberculosis*-mediated repression, could shift the balance toward a suboptimal induction of host immunity. In the current study, we showed that the ability of *M. tuberculosis* to interfere with normal production of IL-12p40 was dependent on its production of mycolic acids with distal chain keto or methoxy groups, and that TDMs containing such modified mycolates could be the effector molecules for this immune evasion mechanism of *M. tuberculosis*. Specifically, our data suggest that *M. tuberculosis* may have evolved keto- and methoxy-modification of the mycolic acids incorporated into TDM in order to manipulate IL-12p40-mediated immunity in the host macrophages. We predicted that the removal of such IL-12p40 inhibitory components of *M. tuberculosis* would lead to a decrease in bacterial burden and would increase host survival. Indeed, Dubnau *et al.* previously showed attenuation of growth of the Δ*mmaA4* mutant (also known as *hma*) in a mouse model of *M. tuberculosis* infection. Extending this observation, our data demonstrated that mice infected with the Δ*mmaA4* mutant survived 400 days, at which time the experiment was terminated. Moreover, the attenuation depended on IL-12p40-mediated immunity. As part of the mechanism of *M. tuberculosis* pathogenesis, our results provide new insights into the link between oxygenated mycolic acids on TDM and the suppression of IL-12p40-mediated immunity contributed by macrophages.

Mycolic acids are long-chain α-alky ß-hydroxy fatty acids unique to mycobacteria, and they comprise approximately 30% of the dry weight of *M. tuberculosis* [34]. Structurally, the mycolic acids can be broadly distinguished into two classes, de-oxygenated (cyclopropyl) and oxygenated (keto and methoxy), based on the chemical functional group at the distal position of their long meromycolate chains. Several cyclopropane synthetases and methyl transferases are involved in the introduction of these functional groups, including the so-called methoxy mycolic acid synthases encoded by the *mmaA3* and *mmaA4* genes that were the focus of our current study. While mycolates are found covalently attached to the arabinogalactan of the *M. tuberculosis* cell wall, they also have an important role as a component of several extractable cell wall glycolipids, such as TMM and TDM. Significantly, both of these glycolipids are shed from the mycobacterium into the cytoplasm of infected macrophages, and they are widely believed to play a role in modulating many of the cellular processes that occur in the *M. tuberculosis*-infected mammalian host [35]. Recently, there has been increased appreciation of the specific bioactivities associated with each functional group. For example, TDM purified from the Δ*cmaA2* mutant of *M. tuberculosis* which lacks *trans*-cyclopropanation of mycolic acids stimulated increased TNF-α production by macrophages [38]. On the other hand, TDM which lacks both *cis*- and *trans*-cyclopropanation, as do those isolated from the Δ*pcaA* mutant, caused delayed TNF-α production [39]. Interestingly, cyclopropanation of mycolic acid affects only TNF-α, but not IL-12p40, production by macrophages.

Our data extend earlier characterizations of the biological activities of TDM and explore the previously uncharacterized role of the distal keto and methoxy groups that are missing from the mycolic acids incorporated in TDMs of the Δ*mmaA4* mutant. Previously, Oswald *et al.* reported that peritoneal macrophages isolated from mice showed activation of transcription of IL-12p40 *ex vivo* when treated with TDM purified from *M. tuberculosis* [40]. The findings of these investigators are consistent with our observation that wild type TDM induced low levels of IL-12p40 production by macrophages. In addition, by comparing the effects of wild type and Δ*mmaA4* TDMs on macrophage production of IL-12p40, we uncovered an underappreciated novel repressing function for wild type TDM. This was evident in the ability of wild type TDM to significantly dampen LPS-induced IL-12p40 production. Since LPS signals mainly through the TLR4 receptor and wild type TDM bioactivity has been found to be independent of TLR4 signaling, it is unlikely that inhibition of LPS-mediated IL-12p40 production resulted from competition between wild type TDM and LPS for the TLR4 receptor [41]. In comparison, Δ*mmaA4* TDM was attenuated in its ability to repress induction of IL-12p40 by LPS, and Δ*mmaA4* TDM was also more directly stimulatory than wild type TDM with regard to activating macrophage production of IL-12p40.

It is likely that the different biological activities observed for TDMs from wild type versus those from Δ*mmaA4* bacteria were based on the chemical and structural differences conferred by the functional groups of their mycolates. In particular, while the lack of methoxy- and keto- groups may have been responsible for the loss of ability to repress the activation of IL-12 transcription, it is also possible that the novel appearance of epoxy mycolates that we observed in the Δ*mmaA4* mutant TDM accounted for at least part of its increased stimulatory effect. Although entirely speculative at this point, this possibility is suggested by the fact that the epoxide functional group is highly reactive, and may thus interact more avidly with cellular components that are normally not engaged during *M. tuberculosis* infection. At present, it is not technically feasible to purify TDMs based on their precise mycolate composition into separate homogeneous groups, and future studies using chemically synthesized TDMs with precisely fixed mycolate structures may be required to clarify the cytokine-inducing or -repressing activity of each mycolic acid functional group when associated with different TDMs.

Previous studies focused on transcriptional profiling of *M. tuberculosis* in the lungs of infected mice have shown that the *mmaA4* gene is upregulated *in vivo*, compared to its level in bacteria growing in culture [42]. This observation suggests the interesting possibility that *M. tuberculosis* remodels its mycolic acid composition as a counter-response to host immunity. In support of this idea, analysis of mycolic acid production during infection has shown that *M. tuberculosis* synthesizes more keto-mycolates following macrophage infection [43]. Evading host immunity by modifying bacterial components that interact with the host is a strategy common among opportunistic bacteria that cause chronic infection. For example, *Pseudomonas aeruginosa* (associated with cystic fibrosis) and *Porphyromonas gingivitis* (associated with periodontal disease) have naturally occurring variants of LPS structures that antagonize cytokine production [44], [45]. Additionally, *Helicobacter pylori* (associated with peptic ulcer disease) flagellin contains natural modifications which allow the bacterium to evade detection by the immune system [46]. A prominent theme emerging from studies of immunologically active glycolipids in *M. tuberculosis* is that this pathogen has evolved a number of mechanisms for modifying these compounds to reduce their recognition by the innate immune system and dampen their tendency to stimulate cytokine production. In addition to the modification of mycolic acids in TDM, the modification of *M. tuberculosis* lipomannan (LM) by attachment of a large arabinan to generate lipoarabinomannan (LAM) also may represent a strategy designed to block the ability of a mycobacterial glycolipid to activate IL-12p40 production [25],[47]. Similarly, the phenol group on phenolic glycolipid alters cytokine production of macrophages. Absence of this phenol group in mutants lacking the *pks 1–15* gene cluster abrogates cytokine-repressing activity, and leads to attenuation of virulence with extended survival in mouse infection studies [48].

Our current findings add to a growing literature demonstrating that *M. tuberculosis* has evolved a repertoire of molecules that disrupt macrophage effector mechanisms. In addition to our current findings for TDM, the ESAT-6 protein of *M. tuberculosis* also suppresses IL-12p40 [49],[50]. This suppression of macrophage IL-12 production is likely to be responsible for increasing bacterial survival during innate immune response, given the critical role of IL-12p40 as a macrophage chemoattractant and in interferon gamma production [51]–[54]. By expressing various IL-12 inhibitors that might function at different times and locales during the course of the infection, *M. tuberculosis* is well adapted to survive even in the face of a normal host immune response. We believed that additional bacterial components of *M. tuberculosis* involved in IL-12p40 repression could be revealed by extending our screening approach to saturation. The identification and removal of mycobacterial genes involved in the inhibition of important cytokine responses, such as we have demonstrated in the current study for *mmaA4*, should provide a straightforward and rational approach for creating more immunogenic strains of attenuated mycobacteria that may ultimately yield more effective vaccines and immunotherapies for the prevention of tuberculosis.

## Materials and Methods

### Bacterial cultures

Cultures of mycobacteria were routinely grown in 7H9-C media which contained Middlebrook 7H9 media supplemented with OADC (oleic acid/albumin/dextrose/catalase) (Difco, Becton-Dickinson), 0.5% Glycerol, and 0.05% Tween-80. Colony morphologies for wild type and mutant *M. tuberculosis* strains were observed by plating bacterial cultures on Middlebrook 7H10 plates supplemented with OADC, 0.5% glycerol, and 0.05% Tween-80. For mutant strains, 50 µg/ml of hygromycin was included in the media.

### Differentiation of bone marrow-derived macrophages and dendritic cells from BALB/c or C57B6 mice

Six-to-eight-week-old female BALB/c mice were purchased from Jackson Laboratory (Bar Harbor, ME). Bone marrow cells were flushed with phosphate buffered saline (PBS) from the femurs of mice and cultured in Dulbecco's Modified Eagle Medium (DMEM), supplemented with 10% heat-inactivated fetal calf serum (FCS) plus 20% conditioned medium from a culture of L929 cells (as a source of M-CSF), for 7 days at 37°C, 5% CO_2_. Bone marrow-derived macrophages were harvested on day 6 to plate for infection with different *M. tuberculosis* strains on day 7. Bone marrow-derived dendritic cells were differentiated by methods described by Lutz, MB *et al.* Briefly, bone marrow cells were seeded at 3×10^5^ cells/100 mm petri dish. The cells were differentiated in the presence of 10 ng/ml GM-CSF (Peprotech). Media were changed every two days. Cells (1.5×10^5^/200 ul media) were seeded into 48 wells on day 7 and infected on day 8.

### Construction of a GFP-based reporter for IL-12p40 expression

The construction of the −350+55 IL-12p40-GFP Raw 264.7 reporter line was described previously [25]. Using a similar strategy to monitor IL-12p40 expression from a full-length (FL) IL-12p40 promoter [55], position −800 to +55 relative to the transcription start site of the IL-12p40 promoter was amplified from C57BL/6 mouse genomic DNA by PCR by using upstream primer 5′ACAGGATTGCACACCTCTTTG 3′ and downstream primer 5′ TTGCTTTGCTGCGAGC3′. The 856 bp PCR product was placed into the TOPO cloning vector (Invitrogen) to create the plasmid pFL.IL-12p40.TOPO. The full-length IL-12p40 GFP reporter construct (pFL.IL-12p40.EGFP-1) was generated by ligating the HindIII/PstI fragment from the pFL.IL-12p40.TOPO into the HindIII and PstI cleaved enhanced green fluorescent protein reporter vector, pEGFP-1 (BD Bioscience). Raw 264.7 cells were stably transfected with pFL.IL-12p40.EGFP-1 using electroporation, as described previously [25]. Following selection with G418 (1 mg/ml), a stable −800+55 IL-12p40-GFP Raw 264.7 macrophage cell line was cloned by limiting dilution under G418 selection and maintained in DMEM with high glucose, supplemented with 10% FCS, 10 mM HEPES, and 1 mg/ml of G418. Clones that express GFP only when treated with LPS (10 ng/ml) or CpG (100uM) were expanded for use.

### Growth of *M. tuberculosis* strains for macrophage infection

For each infection, a new vial of bacterial culture was thawed from stocks kept at −70°C. Thawed *M. tuberculosis* H37Rv, Beijing HN878, or *M. smegmatis* were grown in 10 ml 7H9-C medium, as described above. The Δ*mmaA4* mutant was grown in 7H9-C medium along with 50 µg/ml hygromycin, while the complemented Δ*mmaA4* mutant was grown in 7H9-C medium with 40 µg/ml of kanamycin. All mycobacterial strains were grown to an OD_600 nm_ of between 0.1 and 0.3 prior to infection, since a population of the mycobacterial culture will autolyse when grown to OD ≥0.5 undergo autolysis (data not shown) [56]. Prior to infection, the bacteria were pelleted and resuspended in 7H9-C medium. The resuspended pellets were treated once with 10 sec of continuous sonication to minimize aggregation.

### Mouse infection

Male and female C57BL/6 and IL-12p40^−/−^ mice, 6 to 10 weeks of age, were acquired from Jackson Laboratories. One ml aliquots of frozen suspensions of *M. tuberculosis,* H37Rv, Δ*mmaA4* mutant, or complemented Δ*mmaA4* mutant were thawed and innoculated into 7H9-C media containing the appropriate selecting agents (50 µg/ml hygromycin for Δ*mmaA4*, and 40 µg/ml kanamycin for the complemented mutant). Bacteria from frozen stocks were grown to an OD_600nm_ of between 0.1 and 0.3, and then collected by centrifugation and washed once with PBS- 0.05% Tween-80. Cell pellets were resuspended to 1×10^7^ CFU/ml, and 20 µl of 1∶5 diluted Antifoam (Sigma) was added to 10 ml of the bacteria suspension to prevent froth formation during aerosalization. The bacterial suspension was placed in the nebulizer jar of a whole-body exposure aerosol chamber (Mechanical Engineering Workshop, Madison, WI). Mice were exposed for 20 min, with a chamber purge time of 30 min between strains. 24 hr post-aerosalization, lungs from 3 mice per group were harvested to determine the inoculum per group.

### In vitro mycobacteria infections

Bone marrow-derived macrophages or dendritic cells were seeded in triplicate at 2×10^5^ per well (for macrophages) or 1.5×10^5^ per well (for dendritic cells) in 48-well plates, or 2×10^5^ per well in 96-well plates for the FL.IL-12p40-GFP macrophage reporter cell line. The macrophages were infected with mycobacteria at a multiplicity of infection (MOI) of 3 or 10. After 4 hr incubation in a humidified incubator at 37°C in the presence of 5% CO_2_, non-ingested bacteria were removed by washing gently 3 times with pre-warmed DMEM-C medium for macrophages and with RPMI-C medium for dendritic cells. Each well then received 200 µl DMEM-C or RPMI-C containing 50 µg/ml gentamicin (to kill the remaining extracellular bacteria), and plates were cultured in a humidified incubator at 37°C in the presence of 5% CO_2_.

### ELISA measurement of cytokine production

Infection was allowed to proceed for 16 to 24 hr before cell supernatants were harvested. For time course studies, the supernatants were collected at the additional time points of 48 and 72 hr. Supernatant was filtered with 0.22 µm SpinX columns (Costar) to remove any uningested extracellular bacteria. Cytokines in the conditioned medium were analyzed by sandwich ELISA using the Biosource International (Camarillo, CA) kit for IL-12p40 and TNF-α, following the manufacturer's protocol.

### Flow cytometry

Following infection of the FL.IL-12p40 GFP- reporter macrophage cell line with H37Rv, HN878 Beijing, and *M. smegmatis*, the experiment was allowed to proceed for 16 to 24 hr before processing the cells for FACS analysis. *Mycobacteria* infected cells were trypsinized, fixed with equal volume of 4% paraformaldehyde, and left at 4°C overnight. The following day, GFP expression was ascertained by using the FACSCalibur flow cytometer (BD Biosciences) and analyzed with FlowJo software (Tree Star).

### Construction of *Himar-1* *M. tuberculosis* H37Rv mutant library

The Himar-1 *M. tuberculosis* H37Rv mutant library was generated using the Himar-1 transposon delivered by phage, pHAE159, as described previously [28],[29]. Briefly, the phage-containing mariner transposon was propagated to high titer in MP buffer (50 mM Tris (pH 7.6), 150 mM NaCl, 10 mM MgCl_2_, 2 mM CaC1_2_) and used to transduce the *M. tuberculosis* H37Rv strain. The transductions were plated on 7H10 plates containing 50 µg/ml hygromycin, and placed at 37°C for three weeks. Transductants were picked into 96-well plates containing 200 µl of 7H9 media supplemented with OADC, 0.5% glycerol, 0.05 % Tween-80, and 50 µg/ml hygromycin. A *Himar-1* transposon library of *M. tuberculosis* H37Rv was grown to late-log phase. Aliquots of the mariner *M. tuberculosis* H37Rv library were made into separate 96-well plates for stocking and were diluted and grown to mid-log phase for screening.

### High-throughput screen for *M. tuberculosis* mutants that strongly induce IL-12p40 production

Individual clones from the M. tuberculosis mariner transposon library were grown in wells of 96-well plates with monitoring of cell density by photometric measurements of optical density (OD) at 590 nm using a 96-well plate reader (Victor II plate reader, Perkin Elmer). After 2 days of growth, each well was diluted to approximately 2×10^6^ CFU per 10 µl. The IL-12 reporter strain, Raw 264.7- FL.IL-12p40-GFP, was seeded at 2×10^5^ per well in 96-well plates the day before infection with clones from the mariner transposon library. An aliquot of 10 µl of bacteria from each well was used to infect the FL.IL-12p40-GFP Raw 264 macrophage reporter cell line (i.e., an MOI of 10). After incubation for 4 hr in a 5% CO_2_ humidified incubator at 37°C, non-ingested bacteria were removed by washing gently 3 times with pre-warmed DMEM-C medium. Each well then received 200 µl DMEM-C containing 50 µg/ml gentamicin, and the plates were cultured as before for an additional 16 hr at 37°C, at which time IL-12 expression was found to be maximal. The GFP expression from individual wells on the plate was determined by the use of the Viktor II plate reader using a 488 nm/530 nm excitation/emission filter pair and reading for 1.0 sec per well. For secondary screening of the candidates, the mutants were expanded in 10 ml cultures, and grown to an OD_600nm_ of between 0.1 and 0.3. The IL-12 reporter macrophages were infected with each clone in duplicate and incubated overnight, as described above. After 16 hr, the cells were harvested by trypsinization, and single-cell suspensions from these infected macrophages were generated. An equal volume of 4% paraformaldehyde was added to each well to allow fixation overnight at 4°C, and flow cytometry analysis for GFP expression was performed the following day.

### Mapping of the transposon insertion

Standard genomic DNA (gDNA) preparations were made for transposon insertion mutants. Briefly, 10 ml cultures were grown to an OD_600nm_ of between 0.5 and 0.7, and then centrifuged and the pellets extracted for gDNA. Ten µl aliquots of gDNA were digested with BssHII in 50 µl for 1 hour, after which 4 µl aliquots of digested gDNA were self-ligated for 1 hr using the Rapid Ligation Mixture kit from Roche Laboratories. Five µl of ligation mixture was transformed into competent DH5α pir bacteria and selected for on LB plates containing 150 µg/ml hygromycin. The exact location of each transposon insertion site in the selected mutants was determined by sequencing the flanking *M. tuberculosis* gDNA: Upstream flanking sequence (5′ -AGAATAGACCGAGATAGGGT), Downstream flanking sequence (5′-ACTTTAGATTGATTTCGCGT).

### Construction of phagemid for deletion of *mmaA3* and *mmaA4*

The *mmaA3* (Rv0643c) and *mmaA4* (Rv0642c) mutants were constructed by homologous recombination using specialized transducing phages [29]. The deletion phagemid for the Δ*mmaA3* mutant was constructed by PCR amplification of the 5′-flanking region of *mmaA3* using *M. tuberculosis* H37Rv genomic DNA with the following primer pairs: 0643cRL 5′ TTTTTTTTCCATAGATTGGTCACTCGATCACCGGCTTGCACGTA 3′ and 0643cRR 5′TTTTTTTTCCATCTTTTGGGGAGACGTCGTAGTGCGCTTGGATG 3′. This PCR product was 553 bp. For the 3′ flanking region of *mmaA3*, the following primer pairs were used: 0643c LL 5′TTTTTTTACCATAAATTGGGGAACAGTCGGCGAAGACGGGTTT 3′ and 0643cLR 5′ TTTTTTTTCCATTTCTTGGTGAAGTTGGCCCAGTCGCTCAGCAG 3′. This PCR product was 811 bp.

The deletion phagemid for the Δ*mmaA4* mutant was constructed by PCR amplification of the 5′-flanking region of *mmaA4* from *M. tuberculosis* H37Rv genomic DNA using the primer pairs 0642cRL 5′ TTTTTTTTCCATAGATTGGTTCGAGACGGCGCGTTTCATCA 3′ and 0642cRR 5′ TTTTTTTTCCATCTTTTGGCGACCCGCGTAAGGCAGACCAG 3′ for the 5 prime arm. This PCR product was 994 bp. The primer pairs were 0642cLL 5′TTTTTTTACCATAAATTGGAGCACTCGATCACCGGCTTGCACGTA3′ and 0642cLR 5′TTTTTTTTCCATTTCATGGTCCAACCGCACCCAATGTCCAGCAG 3′ for the downstream arm, which gave rise to a 723 bp PCR product.

Following cloning into p0004S (0642c.p004S or 0643c.p004S), the resulting plasmid was then packaged into the temperature-sensitive phage phAE159, as described earlier, to yield the knockout phages for *mmaA3* (phAE301) and *mmaA4* (phAE302). Specialized transduction was performed, as described previously [29], and the transduction mix was spread on 7H10 plates, selecting with 50 µg/ml hygromycin.

### Confirmation of deletion mutants

Hygromycin-resistant clones were screened for deletion by Southern analysis. Briefly, gDNA from *mmaA3* or *mmaA4* mutants was digested with StuI. Deletion analysis for the Δ*mmaA3* mutant was confirmed by probing the southern blot with the PCR product (811 bp) from the primer pairs 0643c LL & 0643cLR. Following homologous recombination, the *mmaA3* mutant had a 2742 bp fragment as compared to wild type, which gave a 6127 bp fragment. Deletion analysis for the Δ*mmaA4* mutant was confirmed by probing the southern blot with the PCR product (723 bp) from the primer pairs 0642cLL & 0642cRL. Following homologous recombination, the Δ*mmaA4* mutant had a 3542 bp fragment as compared to wild type, which gave a 6127 bp fragment.

### Construction of Δ*mmaA4* complemented strain

Complementation analyses were performed with the cosmid 3E2 (*Rv0630c*–*Rv0654c*), which contained the *mmaA4* gene in the integration-proficient vector pYUB412. The transformation of the mutant strains with the constructs by means of electroporation was described previously. Kanamycin-resistant clones were screened for reversion of mutant colonial morphology.

### Small-scale lipid extraction and MAME analysis

Initially, 10 ml cultures of wild type *M. tuberculosis*, Δ*mmaA4* mutant, or complemented Δ*mmaA4* mutant at an OD _600 nm_∼0.4 were labeled using 1 µCi/ml [^14^C]-acetic acid and further incubated for 12 hr. Cells were recovered by centrifugation at 27,000×g for 10 min and carefully freeze-dried using a Savant SpeedVac. Cellular-associated lipids were extracted twice using 2 ml of CHCl_3_/CH_3_OH/H_2_O (10:10:3, v/v/v) for 3 hr at 50°C. Organic extracts were combined with 1.75 ml CHCl_3_ and 0.75 ml H_2_O, mixed and centrifuged. The lower organic phase was recovered, washed twice with 2 ml of CHCl_3_/CH_3_OH/H_2_O (3:47:48, v/v/v), and then dried and resuspended with 200 µl of CHCl_3_/CH_3_OH (2:1, v/v). The residual cell pellet was subjected to alkaline hydrolysis using 15% aqueous tetrabutylammonium hydroxide (TBAH) at 100°C overnight, followed by the addition of 4 ml of dichloromethane, 300 ml iodomethane, and 4 ml of water. The entire reaction mixture was then mixed for 1 hr. The upper aqueous phase was discarded and the lower organic phase washed twice with water and evaporated to dryness. Mycolic acid methyl esters (MAMES) were re-dissolved in diethyl ether. After centrifugation, the clear supernatant was again dried and resuspended in dichloromethane (100 ml) and an aliquot subjected to 1-dimensional High Performance Thin-Layer Chromatography (1D-HPTLC), using two developments of hexane/ethyl acetate [95:5]). MAMES were visualized by autoradiography by exposure of TLCs to X-ray film (Kodak X-Omat).

### Large-scale lipid extraction and purification of TDM and TMM

Four liter cultures of wild type *M. tuberculosis* or Δ*mmaA4* mutant were grown to OD_600nm_ = 0.4. Mycobacteria were recovered by centrifugation at 3000 RPM for 15 min in a table-top centrifuge. Cellular lipids were extracted twice, as described above, from freeze-dried cells using 200 ml of CHCl_3_/CH_3_OH/H_2_O (10:10:3, v/v/v) for 3 hr at 50°C. Organic extracts were combined with 175 ml CHCl_3_ and 75 ml H_2_O, mixed and centrifuged. The lower organic phase was recovered, washed twice with 200 ml of CHCl_3_/CH_3_OH/H_2_O (3:47:48, v/v/v), dried, and resuspended with 2 ml of CHCl_3_/CH_3_OH (2:1, v/v). The lipid extract was examined by 2-dimensional TLC on aluminum-backed plates of silica gel 60 F_254_ (Merck 5554), using chloroform/methanol/water (100:14:0.8, v/v/v) in the first direction and chloroform/acetone/methanol/water (50:60:2.5:3, v/v/v) in the second direction. TDM and TMM were visualized either by spraying plates with α-naphthol/sulfuric acid, or by spraying with 5 % ethanolic molybdophosphoric acid, followed by gentle charring.

The crude lipid extract (250 mg) dissolved in chloroform/methanol (2:1, v/v) was applied to a diethylaminoethyl (DEAE) cellulose column (2 cm×15 cm) and the flow-through kept for further purification. TDM and TMM were further purified by preparative TLC on 10 cm×20 cm plastic-backed TLC plates of silica gel 60 F254 (Merck 5735, Darmstadt, Germany), run in chloroform/methanol/ammonium hydroxide (80:20:2, v/v/v). The plates were then sprayed with 0.01% 1,6-diphenyl-1,3,5-hexatriene dissolved in petroleum ether/acetone (9:1, v/v), and lipids were visualized under UV light. Following detection, the plates were re-developed in toluene to remove diphenyl-1,3,5-hexatriene, and the corresponding TDM and TMM bands were scraped from the plates and extracted from the silica gel using 3 extractions of chloroform/methanol (2:1, v/v) to provide highly purified TDM and TMM. Quantitation of the purified TDM and TMM was done by directly weighing the material.

The highly purified TDM and TMM from wild type *M. tuberculosis* was reconstituted in petroleum at a concentration of 200 µg/ml. Aliquots of 500 µl were dispensed into endotoxin-free glass vials, and the samples were dried under nitrogen for storage. The TDM and TMM stock was tested for endotoxin contamination using the Limulus Amoebocyte Lysate (LAL) assay from Bio Whittaker, following the manufacturer's protocol. Briefly, the TDM (or TMM) in one of the vials was resuspended in DMSO to a C_f_ = 1 mg/ml. Ten µl of the sample was used in the LAL assay. The TDMs and TMMs from wild type *M. tuberculosis* H37Rv or Δ*mmaA4* mutant were endotoxin-free (data not shown).

### Reconstitution and dilution of TDM or TMM for macrophage stimulation

At the time of the experiment, 100 µg of TDM was reconstituted in 500 µl of petroleum ether. A series of 2-fold dilutions of TDM was made with petroleum ether to yield 10 µg/100 µl, 5 µg/100 µl, and 2.5 ug/100 µl, after which 100 µl of each dilution was used to coat a 48-well plate. The plate was air dried to evaporate the solvent, washed once with PBS, and then air dried again. The TDM dose used in this assay was higher than that typically used for pathogen glycolipids from gram-negative bacteria such as LPS, but comparable to that used for glycolipids and other cell wall-associated immune activators of gram-positive bacteria, such as lipoteichoic acid and peptidoglycan [57]–[59]. Bone marrow-derived macrophages were then immediately added at 2×10^5^ cells/200 µl per well in a 48-well plate. For wild type TDM and Δ*mmaA4* TDM cotreatment, 10 µg of wild type TDM and 5 µg of *mmaA4* TDM were added in 100 µl each of petroleum ether to the same wells of a 48-well plate. The contents were mixed to ensure even distribution of the lipids before the plate was air-dried, washed with PBS, and then air dried again, before the addition of macrophages. For *E. coli* LPS and wild type TDM cotreatment, the wells were first coated with 5 µg wild type TDM, air dried, washed with PBS, and then air dried again. This was followed by the addition of bone marrow-derived macrophages and, 16 hr later, 100 ng/ml *E. coli* LPS. Culture supernatants were harvested, filtered, and then analyzed by ELISA for cytokine levels, as described above. TDM from *M. tuberculosis* purchased from Sigma was also tested. The IL-12p40 response of macrophage and dendritic cells is similar to that of TDM purified by us from wild type *M. tuberculosis.* TDM studies reported in Fig. S2 was purchased from Sigma.

Supplementary materials and methods for total lipid extraction and analysis can be found in Protocol S1.

### Accession numbers

mmaA4 METHOXY MYCOLIC ACID SYNTHASE 4 (HYDROXY MYCOLIC ACID SYNTHASE) [*Mycobacterium tuberculosis H37Rv*] GeneID: 888056

mmaA3 METHOXY MYCOLIC ACID SYNTHASE 3 [*Mycobacterium tuberculosis H37Rv*] GeneID: 1091772

ackA ACETATE/PROPIONATE KINASE [*Mycobacterium tuberculosis H37Rv*] GeneID: 886399

Rv3435c PROBABLE CONSERVED TRANSMEMBRANE PROTEIN [*Mycobacterium tuberculosis H37Rv*] GeneID: 887564

## Supporting Information

Figure S1 Increased induction of IL-12p40 and TNF-α by the Δ*mmaA4 M. tuberculosis* mutant in C57B6 bone marrow-derived macrophages. Bone marrow-derived macrophages from C57BL/6 mice were infected with wild type *M. tuberculosis* H37Rv or the Δ*mmaA4* mutant at an MOI of 10, or left untreated (UT). Conditioned media from macrophages were harvested at 24 hr post-infection. IL-12p40 and TNF-α production were determined by ELISA. (UT) untreated. (*) undetectable levels. Values are statistically significant between wild type and Δ*mmaA4* mutant; **, p<0.01; ***, p<0.001 (one-way ANOVA, Bonferroni post-tests). Values are the means±SD of triplicate samples and are representative of 2 separate experiments.
(0.92 MB TIF)

Figure S2 Production of IL-12p40 in dendritic cells infected with bacteria or TDM. (A) Bone marrow-derived dendritic cells from Balb/c were infected with wild type *M. tuberculosis* H37Rv or the Δ *mmaA4* mutant at an MOI of 10, or left untreated (UT). Conditioned media were harvested at 24 hr post-infection. IL-12p40 production was determined by ELISA. (B) Bone marrow-derived dendritic cells from Balb/c were treated with either 10 ug of TDM from *M. tuberculosis* or 50 ng/ml of lipopolysaccharide (LPS). Conditioned media were harvested at 24 hr post-treatment. IL-12p40 production was determined by ELISA.
(0.47 MB TIF)

Figure S3 Analysis of mycolic acids from mycobacteria and TDM prep. (A) Schematic representation of α-, methoxy-, and keto-mycolic acids synthesized by wild type *M. tuberculosis* H37Rv strain. (B) Thin-layer chromatographic analysis of lipids extracted from [14C] acetate-labeled cultures of wild type *M. tuberculosis* H37Rv, the Δ*mmaA4* mutant, and the complemented Δ*mmaA4*. The cultures were grown to mid-exponential phase in 7H9 containing 0.05% Tween-80 media, at which time [14C] acetate was added, and they were incubated for an additional 12 hr. Lipids were then extracted from cultures for analysis. MAMEs were prepared and analyzed by 1D-High Performance Thin-Layer Chromatography (1D-HPTLC), using two developments of hexane/ethyl acetate [95:5] and visualized by autoradiography. (1) and (3) wild type *M. tuberculosis* H37Rv; (2) Δ*mmaA4* mutant; (4) Δ*mmaA4* mutant complemented. (C) Thin-layer chromatography of purified TDM from *M. tuberculosis* wild type and Δ*mmaA4* mutant developed with chloroform/methanol/water (90:10:1, vol/vol/vol). (1) Wild type *M. tuberculosis* H37Rv; (2) Δ*mmaA4* mutant.
(2.19 MB TIF)

Figure S4 Major extractable lipids from wild type *M. tuberculosis* H37Rv and Δ*mmaA4* mutant. Apolar and polar lipids from wild type and mutant bacteria, including phthiocerol dimycocerosates (PDIMs), sulfolipids, trehalose dimycolates (TDMs), glucose monomycolates (GMMs), and phospholipids, were unaltered in their quantities and TLC mobilities. 2D Thin-layer chromatographic analysis of lipids extracted from [14C] acetate-labeled cultures of wild type *M. tuberculosis* H37Rv or the Δ*mmaA4* mutant. (A) Apolar lipid extracts, run with solvent systems A–D. (B) Polar lipid extracts, run with solvent systems D and E. See Protocol S1 for description of solvent systems. Lipids were visualized by phosphorimaging and compared to known standards. (?) unknown.
(4.61 MB TIF)

Figure S5 Macrophages treated with trehalose monomycolate of wild type *M. tuberculosis* (wtTMM) produced less IL-12p40 and TNF-α than those treated with trehalose monomycolate from Δ*mmaA4* mutant (*mmaA4*TMM). Bone marrow-derived macrophages were treated with wtTMM or Δ*mmaA4* TMM. Supernatants were analyzed for the presence of IL-12p40 and TNF-α by ELISA. Vehicle treatment was the solvent in which the TMM was dissolved. Values were statistically significant between wild type and the Δ*mmaA4* mutant; ***, p<0.001 (one-way ANOVA, Bonferroni post-tests). (*) Undetectable levels. (UT) = vehicle solvent. Values are the means±SD of triplicate samples and are representative of two separate experiments performed on two independent batches of purified TMM from wild type *M. tuberculosis* H37Rv or Δ*mmaA4* mutant.
(0.83 MB TIF)

Protocol S1 Supplementary materials and methods.
(0.03 MB DOC)
