# Supplementary figures and images for: Mycolic Acid Modification by the mmaA4 Gene of M. tuberculosis Modulates IL-12 Production
Source: PLoS Pathog. 2008 Jun 6;4(6):e1000081. doi: 10.1371/journal.ppat.1000081 (PMC2390761; doi:10.1371/journal.ppat.1000081)

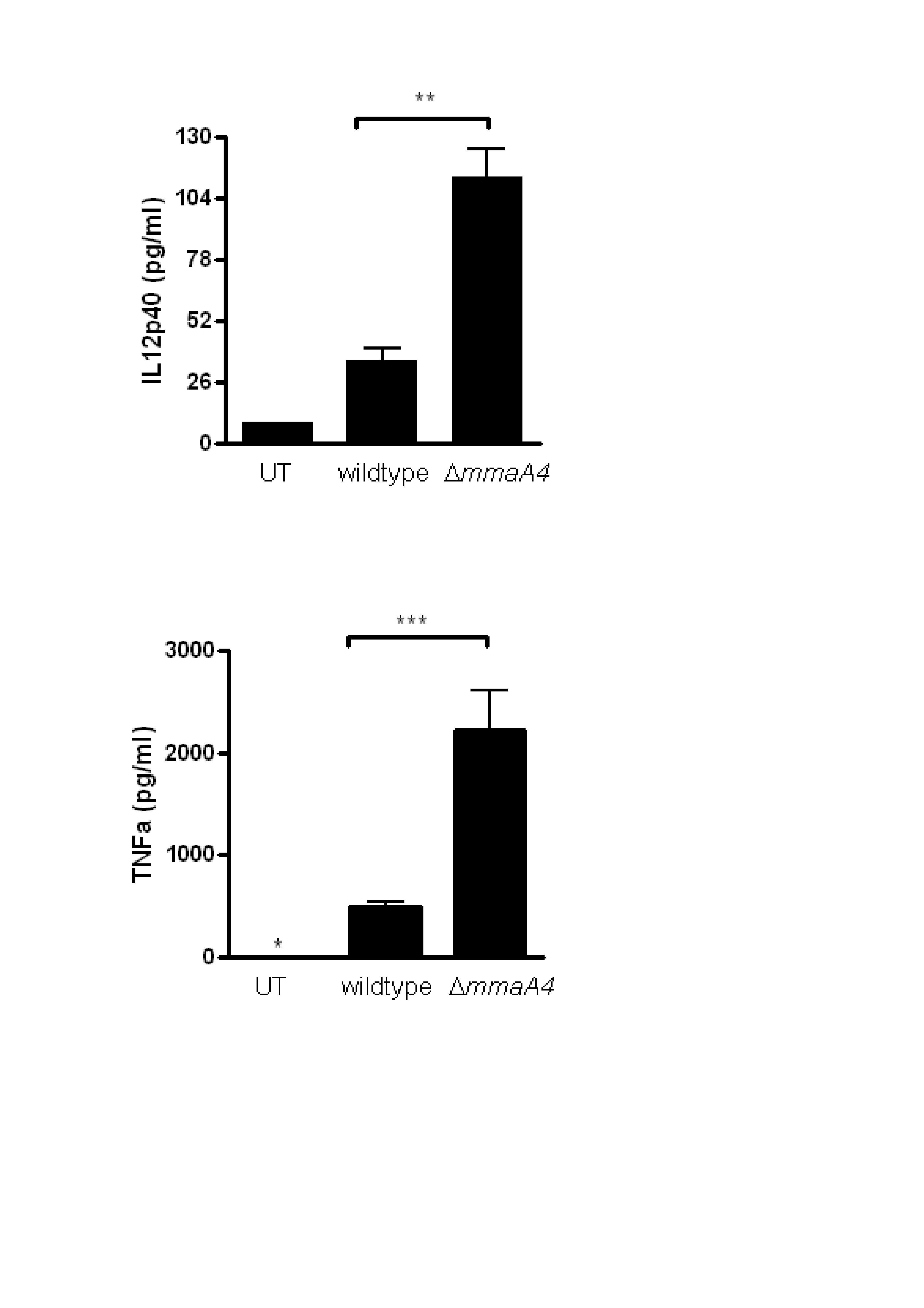

Supplement: Figure S1 — Increased induction of IL-12p40 and TNF-α by the ΔmmaA4 M. tuberculosis mutant in C57B6 bone marrow-derived macrophages. Bone marrow-derived macrophages from C57BL/6 mice were infected with wild type M. tuberculosis H37Rv or the ΔmmaA4 mutant at an MOI of 10, or left untreated (UT). Conditioned media from macrophages were harvested at 24 hr post-infection. IL-12p40 and TNF-α production were determined by ELISA. (UT) untreated. (*) undetectable levels. Values are statistically significant between wild type and ΔmmaA4 mutant; **, p<0.01; ***, p<0.001 (one-way ANOVA, Bonferroni post-tests). Values are the means±SD of triplicate samples and are representative of 2 separate experiments. (0.92 MB TIF) [file ppat.1000081.s001.tif]

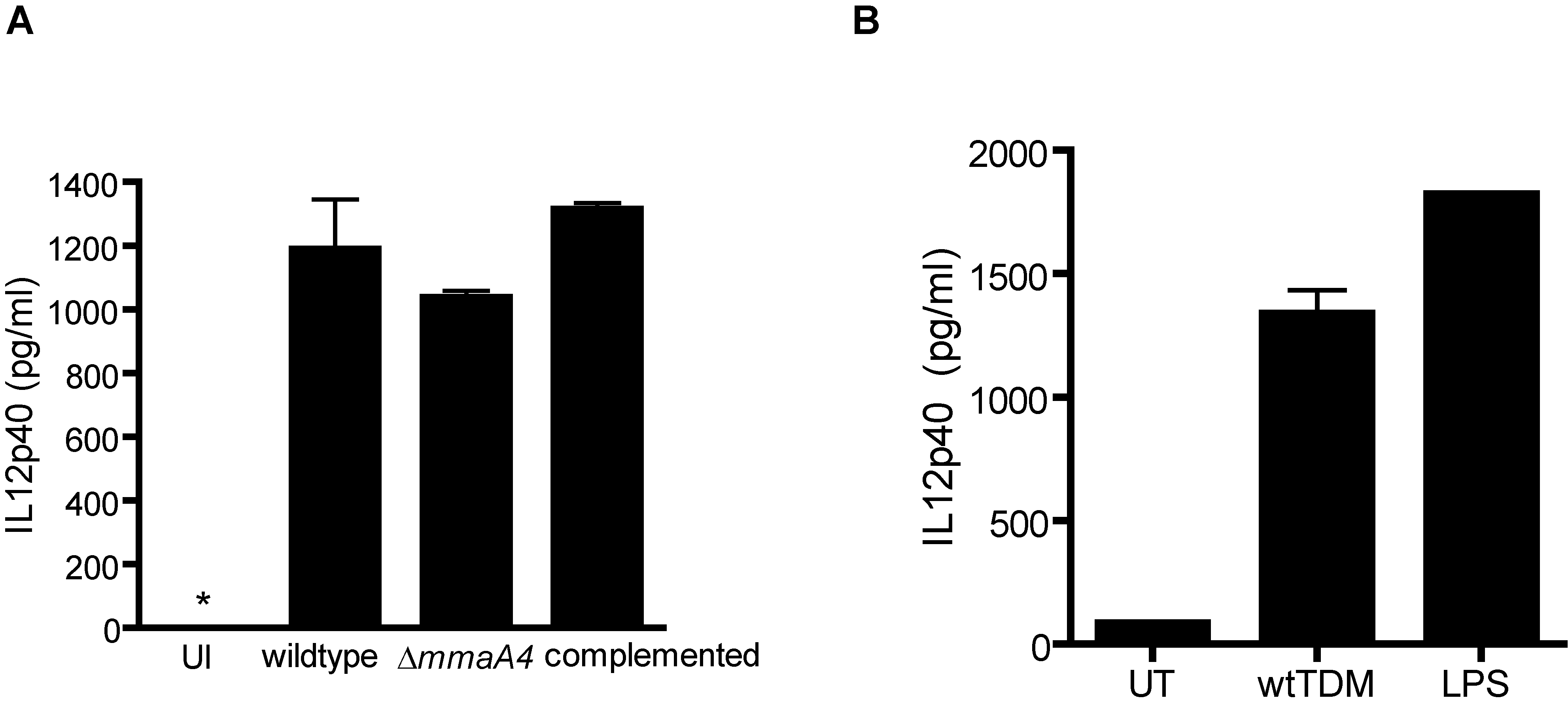

Supplement: Figure S2 — Production of IL-12p40 in dendritic cells infected with bacteria or TDM. (A) Bone marrow-derived dendritic cells from Balb/c were infected with wild type M. tuberculosis H37Rv or the Δ mmaA4 mutant at an MOI of 10, or left untreated (UT). Conditioned media were harvested at 24 hr post-infection. IL-12p40 production was determined by ELISA. (B) Bone marrow-derived dendritic cells from Balb/c were treated with either 10 ug of TDM from M. tuberculosis or 50 ng/ml of lipopolysaccharide (LPS). Conditioned media were harvested at 24 hr post-treatment. IL-12p40 production was determined by ELISA. (0.47 MB TIF) [file ppat.1000081.s002.tif]

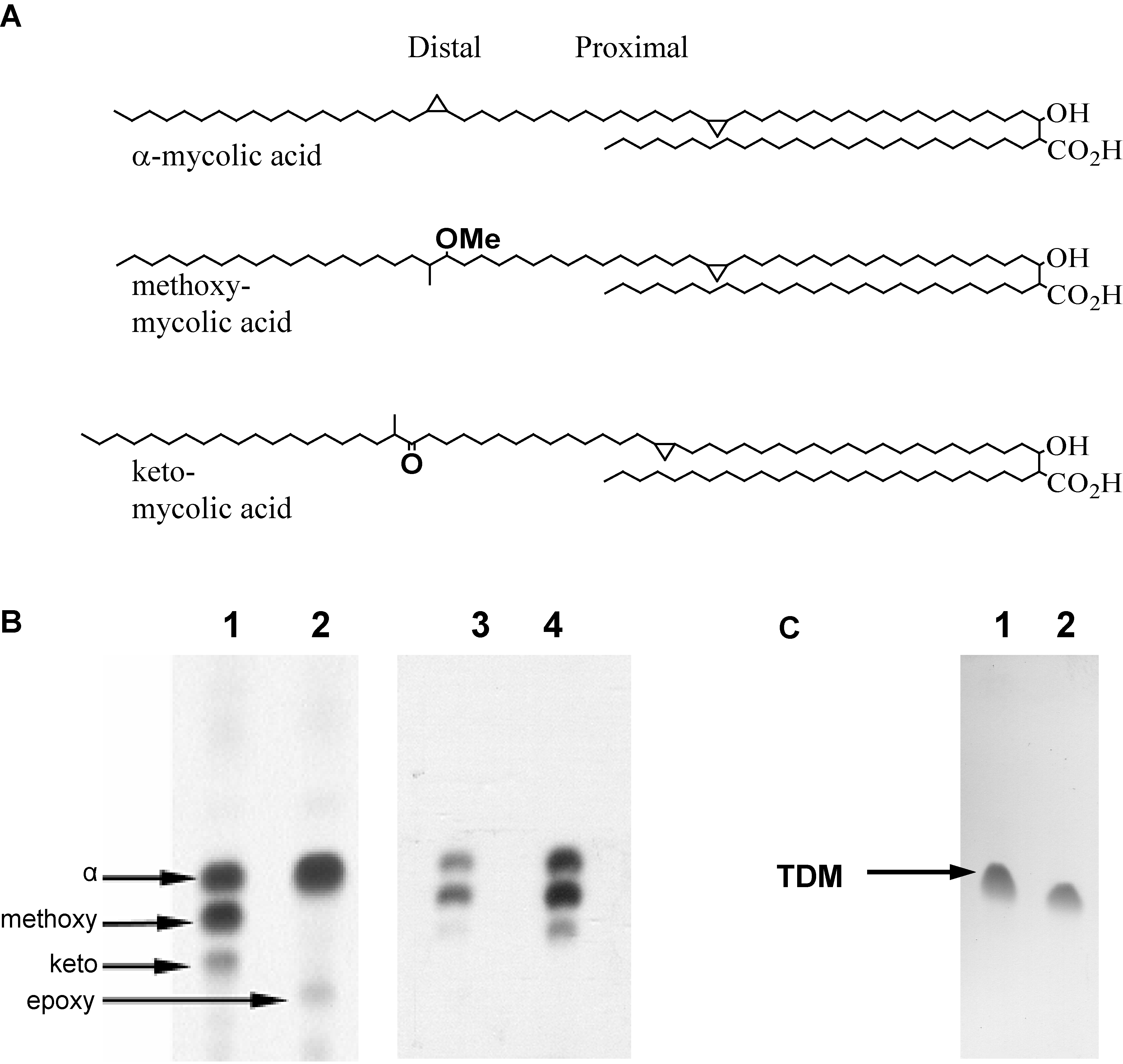

Supplement: Figure S3 — Analysis of mycolic acids from mycobacteria and TDM prep. (A) Schematic representation of α-, methoxy-, and keto-mycolic acids synthesized by wild type M. tuberculosis H37Rv strain. (B) Thin-layer chromatographic analysis of lipids extracted from [14C] acetate-labeled cultures of wild type M. tuberculosis H37Rv, the ΔmmaA4 mutant, and the complemented ΔmmaA4. The cultures were grown to mid-exponential phase in 7H9 containing 0.05% Tween-80 media, at which time [14C] acetate was added, and they were incubated for an additional 12 hr. Lipids were then extracted from cultures for analysis. MAMEs were prepared and analyzed by 1D-High Performance Thin-Layer Chromatography (1D-HPTLC), using two developments of hexane/ethyl acetate [95:5] and visualized by autoradiography. (1) and (3) wild type M. tuberculosis H37Rv; (2) ΔmmaA4 mutant; (4) ΔmmaA4 mutant complemented. (C) Thin-layer chromatography of purified TDM from M. tuberculosis wild type and ΔmmaA4 mutant developed with chloroform/methanol/water (90:10:1, vol/vol/vol). (1) Wild type M. tuberculosis H37Rv; (2) ΔmmaA4 mutant. (2.19 MB TIF) [file ppat.1000081.s003.tif]

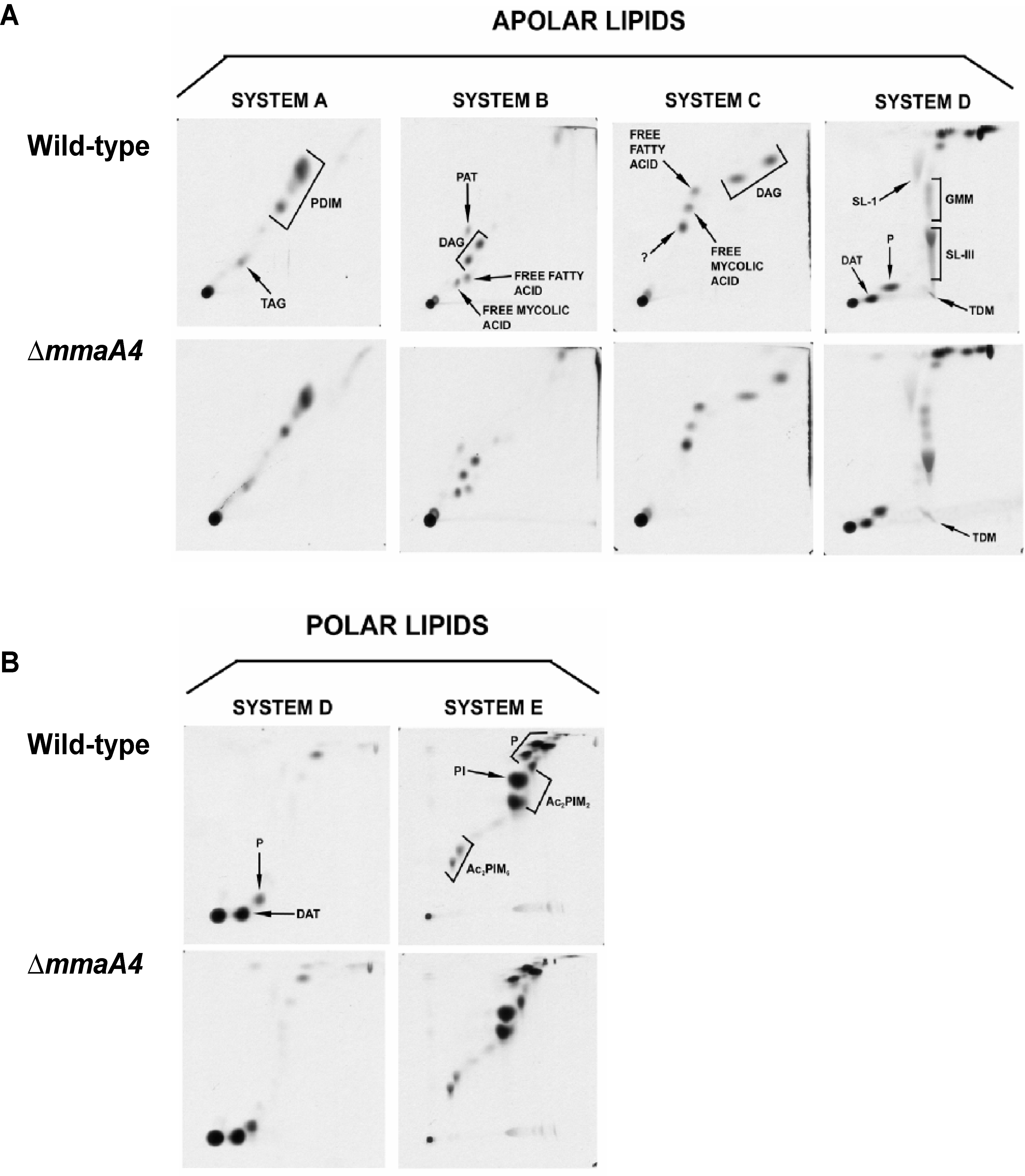

Supplement: Figure S4 — Major extractable lipids from wild type M. tuberculosis H37Rv and ΔmmaA4 mutant. Apolar and polar lipids from wild type and mutant bacteria, including phthiocerol dimycocerosates (PDIMs), sulfolipids, trehalose dimycolates (TDMs), glucose monomycolates (GMMs), and phospholipids, were unaltered in their quantities and TLC mobilities. 2D Thin-layer chromatographic analysis of lipids extracted from [14C] acetate-labeled cultures of wild type M. tuberculosis H37Rv or the ΔmmaA4 mutant. (A) Apolar lipid extracts, run with solvent systems A–D. (B) Polar lipid extracts, run with solvent systems D and E. See Protocol S1 for description of solvent systems. Lipids were visualized by phosphorimaging and compared to known standards. (?) unknown. (4.61 MB TIF) [file ppat.1000081.s004.tif]

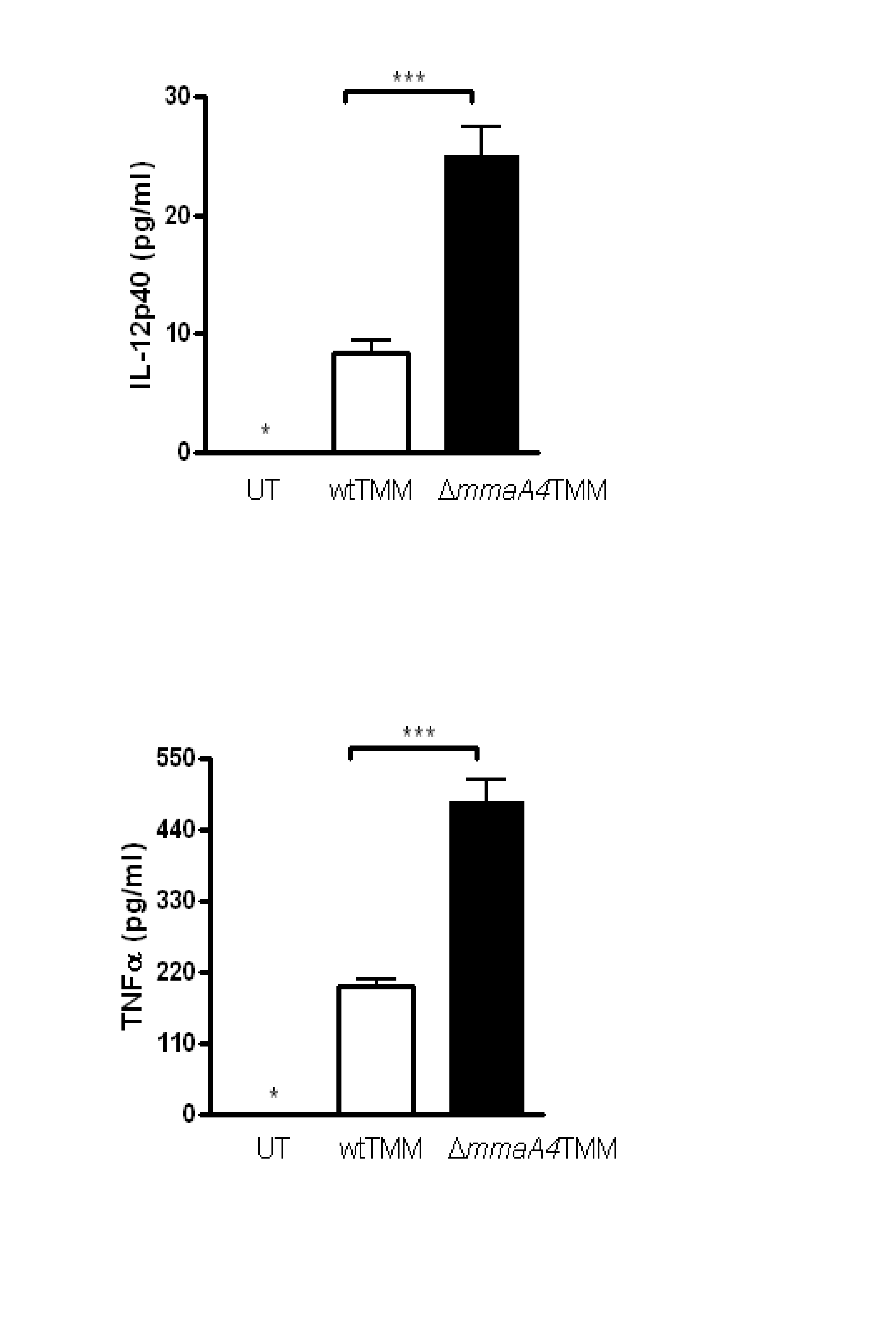

Supplement: Figure S5 — Macrophages treated with trehalose monomycolate of wild type M. tuberculosis (wtTMM) produced less IL-12p40 and TNF-α than those treated with trehalose monomycolate from ΔmmaA4 mutant (mmaA4TMM). Bone marrow-derived macrophages were treated with wtTMM or ΔmmaA4 TMM. Supernatants were analyzed for the presence of IL-12p40 and TNF-α by ELISA. Vehicle treatment was the solvent in which the TMM was dissolved. Values were statistically significant between wild type and the ΔmmaA4 mutant; ***, p<0.001 (one-way ANOVA, Bonferroni post-tests). (*) Undetectable levels. (UT) = vehicle solvent. Values are the means±SD of triplicate samples and are representative of two separate experiments performed on two independent batches of purified TMM from wild type M. tuberculosis H37Rv or ΔmmaA4 mutant. (0.83 MB TIF) [file ppat.1000081.s005.tif]
