# Supplementary material for: Mycolic Acid Modification by the mmaA4 Gene of M. tuberculosis Modulates IL-12 Production
Source: PLoS Pathog. 2008 Jun 6;4(6):e1000081. doi: 10.1371/journal.ppat.1000081 (PMC2390761; doi:10.1371/journal.ppat.1000081)
Supplement: Protocol S1 — Supplementary materials and methods. (0.03 MB DOC) [file ppat.1000081.s006.doc]

**Protocal S1**

**Extraction and analysis of free lipids***.* Cells were harvested by centrifugation, washed once with PBS, and a small-scale lipid extraction performed. Initially, 2 ml of CH_3_OH:0.3% NaCl (100:10) and 1 ml of petroleum ether (bp 60–80) were added to each tube and allowed to mix for 20 min. The tubes were then centrifuged at 3500 rpm for 5 min, and the upper phase was removed and placed into a pre-weighed tube. The lower layer was re-extracted using 1 ml of petroleum ether (bp 60-80), allowed to mix for a further 20 min, and centrifuged. The resulting upper layer was removed, and the two upper layers were pooled and dried under nitrogen to yield the apolar lipid fraction. The lower aqueous methanol extract was then combined with 2.3 ml of CHCl_3_:CH_3_OH:H_2_O (90:100:30, v/v/v) [ok, here and many places below in this section?] and allowed to mix for 1 hr. The tube was then centrifuged at 3500 rpm for 5 min and the supernatant removed and placed into a pre-weighed tube. The residue was re-extracted using 0.75 ml of CHCl_3_:CH_3_OH:H_2_O (50:100:40, v/v/v) and allowed to mix for 30 min. The tube was then centrifuged at 3500 rpm for 5 min and the supernatant removed and combined with the earlier organic extract. The residue was again re-extracted using 0.75 ml of CHCl_3_:CH_3_OH:H_2_O (50:100:40, v/v/v) and allowed to mix for 30 min. The tube was then centrifuged at 3500 rpm for 5 min and the supernatant removed and combined with the 2 earlier organic extracts and mixed with 1.3 ml of CHCl_3_ and 1.3 ml of 0.3% NaCl. The combined extracts were allowed to mix for 20 min, and the tubes were then centrifuged at 3500 rpm for 5 min. The lower organic layer was removed and placed into a pre-weighed tube and dried under nitrogen to afford the polar lipid fraction. The apolar and polar lipid extracts were resuspended in CHCl_3_:CH_3_OH (2:1, v/v) and crude lipid (50,000 cpm) was applied to the corners of 6.6 x 6.6 cm pieces of Merck 5554 aluminum-backed TLC plates. The plates were developed in a series of solvent systems, designed to cover the whole range of lipid polarities, as described [1]. For apolar lipid extracts, these systems were named systems A – D, and for polar lipid extracts, systems D and E. System A TLCs were run 3 times in direction 1 (petroleum ether 60-80: ethyl acetate 98:2) and once in direction 2 (petroleum ether 60-80: acetone 98:2). System B TLCs were run 3 times in direction 1 (petroleum ether 60-80: acetone 92:8) and once in direction 2 (toluene: acetone 95:5). Systems C, D, and E TLCs were run once in each direction. Solvents used for system C were CHCl_3_:CH_3_OH (96:4, v/v) in the first direction and toluene: acetone (80:20) in the second. Solvents for system D were CHCl_3_:CH_3_OH:H_2_O (100:14:0.8, v/v/v) in the first direction and chloroform:acetone:CH_3_OH:H_2_O (50:60:2.5:3, v/v/v) in the second. Solvents for system E were CHCl_3_:CH_3_OH:H_2_O (60:30:6, v/v/v) in the first direction and CHCl_3_:acetic acid:CH_3_OH:H_2_O (40:25:3:6, v/v/v) in the second. Lipids were visualized by phosphorimaging (Kodak K Screen) and compared to known standards [1].

1. Besra GS (1998) Preparation of cell wall fractions. Stoker TPaNG, editor. Totowa, NJ: Humanna Press. p. 91-107
